# Mechanical regulation of bone remodeling

**DOI:** 10.1038/s41413-022-00190-4

**Published:** 2022-02-18

**Authors:** Lijun Wang, Xiuling You, Lingli Zhang, Changqing Zhang, Weiguo Zou

**Affiliations:** 1 Institute of Microsurgery on Extremities, Department of Orthopedic Surgery, Shanghai Jiao Tong University Affiliated Sixth People’s Hospital, 200233 Shanghai, China; 2 State Key Laboratory of Cell Biology, Shanghai Institute of Biochemistry and Cell Biology, CAS Center for Excellence in Molecular Cell Science, Chinese Academy of Sciences, University of Chinese Academy of Sciences, 320 Yueyang Road, 200031 Shanghai, China

**Keywords:** Bone, Bone quality and biomechanics

## Abstract

Bone remodeling is a lifelong process that gives rise to a mature, dynamic bone structure via a balance between bone formation by osteoblasts and resorption by osteoclasts. These opposite processes allow the accommodation of bones to dynamic mechanical forces, altering bone mass in response to changing conditions. Mechanical forces are indispensable for bone homeostasis; skeletal formation, resorption, and adaptation are dependent on mechanical signals, and loss of mechanical stimulation can therefore significantly weaken the bone structure, causing disuse osteoporosis and increasing the risk of fracture. The exact mechanisms by which the body senses and transduces mechanical forces to regulate bone remodeling have long been an active area of study among researchers and clinicians. Such research will lead to a deeper understanding of bone disorders and identify new strategies for skeletal rejuvenation. Here, we will discuss the mechanical properties, mechanosensitive cell populations, and mechanotransducive signaling pathways of the skeletal system.

## Introduction

The skeleton effectively supports the movements of the body and is thus continuously subjected to various environmental forces. In 1892, Julius Wolff proposed that bone density adapts to the mechanical forces placed on the bone.^1^ Much later, Harold Frost clarified this observation and described a control circuit called the ‘mechanostat’ that links the degree of strain induced by mechanical forces to skeletal remodeling.^2^ Long-term high-intensity exercises, for example, strengthen bone in the supporting part of the body. In contrast, long exposure to microgravity can lead to a monthly loss of 1%–1.5% of volumetric bone mineral density in the weight-bearing bones due to increased bone resorption and decreased bone formation.^3,4^ In addition, long-term bed rest and paralysis can substantially reduce the mechanical stress placed on the bone, thus resulting in disuse osteoporosis.^5^ The progress made in skeletal mechanobiology can lead to a deeper understanding of bone disorders and identify new strategies for skeletal regeneration. Accordingly, in this review, we will discuss the effects of mechanical forces on bone homeostasis and highlight mechanosensitive signaling pathways.

## Mechanosensitive cells

**Fig. 1:**
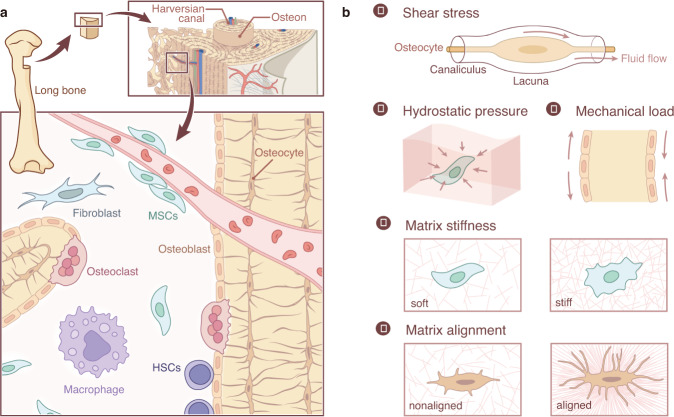
The structural basis of mechanical stress in skeletal cells. **a** The skeletal system contains osteoblasts, osteoclasts, osteocytes, and their progenitors, all sensitive to mechanical stimuli. Osteocytes are the most common mechanical sensors among these cells due to their structure and location in the bone matrix. Mesenchymal stem cells and osteoblast progenitors can sense the FSS in the bone marrow cavity and the strain on the bone. Osteoclasts are both mechanosensitive cells and the effectors for other mechanosensitive cells. **b**. The mechanical stimuli placed on bone may include shear stress, hydrostatic pressure, mechanical stretch and tension, matrix stiffness, and matrix alignment.

The bone is composed of approximately 10% cells, 60% minerals, and 30% organic matter. This composition can effectively maintain the mechanical properties of bone.^6^ Mature bone consists primarily of trabecular bone and cortical bone. The trabecular bone is mainly located in the epiphysis and inside the flat bone. The direction of the trabecular bone structure is consistent with the main stress trace of the bone, and its arrangement and thickness are adjusted to provide optimal load-bearing properties. Cortical bone is a dense external layer of bone that is critical for body structure and weight-bearing due to its high resistance to bending and torsion. In humans, cortical bone is composed of cylindrical structures called osteons. The osteon comprises a central canal filled with nerves and blood vessels surrounded by concentric lamellae. Circumferential lamellae are inserted between the osteons. Osteocytes are buried within fibrous mineralized lamellae. Rather than mineralizing, osteocytes form small chambers (lacunae) and fluid-filled pipes (canaliculi) that provide a structural basis for the generation of fluid shear stress (FSS). Movement and other mechanical forces can result in FSS, bone deformation, and modulation of the alignment of bone extracellular matrix (Fig. 1a). Because of the dynamics and complexity of mechanical forces, it is difficult to observe the reaction of cells to specific forces in vivo. To circumvent this difficulty, researchers have designed in vitro systems to allow the application of several kinds of mechanical variables, including FSS, hydrostatic pressure, mechanical load, matrix stiffness, and matrix topology, and to investigate subsequent cellular behavior (Fig. 1b). Here, we will discuss the current understanding of mechanosensitive cells and their mechanical environments.

Osteocytes constitute 85%–90% of all adult bone cells. These cells sense mechanical stimuli through their cell bodies, dendrites, and cilia and can then transmit signals through cellular dendrites and secreted proteins in an autocrine and paracrine manner.^7–9^ The mechanosensitive characteristics of osteocytes in the regulation of bone homeostasis have been discussed in detail elsewhere.^10,11^ During mechanical loading, poroelastic interactions within the lacunar-canalicular system (LCS) result in fluid flow that is primarily sensed by osteocytes. In addition to fluid flow in the LCS, dynamic intramedullary pressurization results in fluid flow in the marrow cavity and in the endosteal surface, potentially exposing bone marrow mesenchymal stem cells (BMSCs) and bone lining cells to fluid flow. Modulating intramedullary pressure can enhance the structural adaptation of trabecular bone in mice with ablated osteocytes.^12^ These mice have been shown to be resistant to unloading-induced bone loss, providing strong evidence for the role of osteocytes in mechanotransduction.^13^ Osteoclast activity was also increased in these mice due to upregulated expression of RANKL, a cytokine associated with osteoclast development.^13^ Recently, osteoclasts were shown to sense the damage-associated molecular patterns (DAMPs) released by necrotic osteocytes.^14^ Thus, mechanical unloading-associated bone loss could be a result of crosstalk between osteocytes and osteoclasts.

Multiple mechanical forces can drive the proliferation and differentiation of BMSCs.^15,16^ According to several in vitro studies,^17–19^ mechanical forces can also induce substantial changes in the osteoblast cytoskeleton, cell and nucleus morphology, and volume. FSS increased the expression of osteogenic genes in mesenchymal stem cells.^20,21^ Similarly, oscillatory fluid flow led to specific gene expression in BMSCs in a manner dependent on the magnitude, frequency, and duration of shear stress. Short-term fluid flow stimulation promoted the expression of *Cox2*, *Opn*, and *Runx2* in the early stage of osteogenesis, while long-term stimulation increased the formation of collagen and matrix in the late stage.^17^

Osteoclasts are also mechanosensitive cells. FSS has been reported to affect the cell morphology and gene expression of osteoclasts and to inhibit their differentiation without affecting their viability.^22–24^ FSS also stimulates nitric oxide (NO) and prostaglandin E_2_ (PGE2) production in bone marrow-derived preosteoclast-like cells.^25^ Studies of ion channels also provide evidence of the mechanosensitive capabilities of osteoclasts. STIM1 and TRPV4 are Ca^2+^ channels that are highly expressed in the early and late stages, respectively, of osteoclast differentiation. Blockage of STIM1 or TRPV4 at these respective stages caused a reduction in FSS-induced Ca^2+^ oscillations to almost undetectable levels, thus indicating that STIM1 and TRPV4 may act as mechanosensitive ion channels for osteoclasts.^26^ Another recent study showed that fluid flow-derived mechanical stimuli can enhance or suppress the formation of osteoclasts from hematopoietic progenitor cells in a manner dependent on the shear stress rate, amplitude, and duration.^27^ These observations indicate that osteoclasts and their progenitors can respond to specific mechanical stresses in the bone niche (Fig. 1a). Together, these data demonstrate that bone possesses multiple mechanosensitive cellular populations. The activity of these cells in response to mechanical stimuli can have significant effects on the skeletal system.

## Mechanosensitive structures

**Fig. 2:**
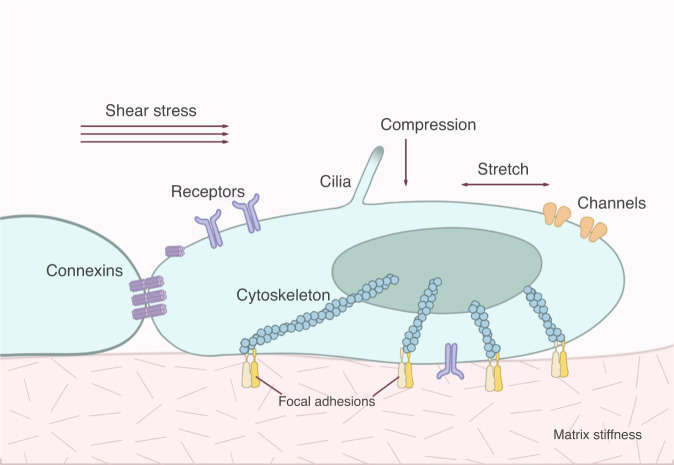
Forces and cellular structures involved in mechanosensation. Mechanical stresses of varying type and intensity are sensed by different families of cellular structures, including integrins, receptors, ion channels, connexins, and cilia.

Mechanoreceptors can sense a variety of external and internal mechanical forces. The detection of external mechanical signals requires mechanoreceptors to be in direct contact with the external environment or to sense changes in the intermediate media, such as those in cell membranes caused by pressure and shear forces. Various cell surface proteins or membrane structures, including integrins, ion channels, connexons, G-protein coupled receptors, and primary cilia, are considered potential mechanosensitive structures (Fig. 2). These structures share certain common features: first, the structures can directly sense single or multiple mechanical forces; second, these mechanical forces can modulate the conformation or activity of structures to activate downstream signaling pathways and direct cell behaviors. Below, we discuss these mechanosensitive structures, downstream signaling pathways, and corresponding cell behaviors in the skeletal system. All molecules and animal models discussed in this review are listed in Tables 1 and 2.

**Table 1:** Mechanosensors reported by in vitro or in vivo studies

Type of study	Molecule	Cell type	Reference
in vitro	αvβ3	MLO-Y4	^46^
in vitro	β1	MG-63 cells	^47,48^
in vivo	Cilium		^83,86^
in vitro	Cilium	MC3T3-E1/MLO-Y4	^87^^,^^88^
in vitro	CX43	ROS 17/2.8 cell line	^68^
in vitro	CX43	MLO-Y4	^19,69^
in vivo	CX43		^70,71,73–75,77^
in vitro	FAK		^45^
in vitro	MACF1	MC3T3-E1	^149,150,151^
in vivo	OPN		^53^
in vitro	PTH1R	MC3T3-E1	^103^
in vivo	PIEZO1/2		^121–123,125^
in vitro	STIM1	osteoclasts	^26^
in vitro	TRPV4	osteoclasts	^26,140^
in vivo	TRPV4	osteoclasts	^137–139^
in vitro	TRPV4	MSCs	^132–134^
in vivo	TRPV4		^127^

**Table 2:** Mouse bone phenotypes resulting from specific gene knockouts (KO) or mutations

Mice	Phenotype	Reference
Conditional osteocyte ablation (DMP1-Diphtheria toxin receptor)	Bone loss with defective mechanotransduction.	^12,13^
Conditional deletion of integrin β1 in osteoblasts (Col1 (2.3 kb)-Cre; β1^*fl/fl*^ CKO)	Normal in the physiological state; resist bone loss and increase whole bone stiffness and strength in the hindlimb unloading model.	^52^
OPN KO	Slight bone loss; osteoblasts and osteoclasts are not altered in the unloading condition.	^53^
Cx43 KO	Osteoblast dysfunction, delayed mineralization, and craniofacial abnormalities.	^70^
Conditional deletion of Cx43 in osteoblasts (Col1 (2.3 kb)-Cre; Cx43^*fl/fl*^ CKO)	Bone loss, osteoblast function impairment, and reduction in anabolic response to mechanical loading in vivo.	^73^
Conditional deletion of Cx43 in osteocytes (Dmp1 (8 kb)-Cre; Cx43^*fl/fl*^ CKO)	Bone loss and osteoblast function impairment; enhanced periosteal bone formation rate and mineral apposition rate; higher mineralizing surface in response to mechanical loading.	^72^
Conditional deletion of Cx43 in osteocytes (Ocn-Cre; Cx43^*fl/fl*^ CKO)	Increased bone resorption through regulation of the RANKL/OPG ratio; enhanced anabolic response to mechanical loading.	^74^
Conditional deletion of Cx43 in osteocytes (Ocn-Cre; Cx43^*fl/fl*^ CKO)	Preserved trabecular bone mass and cortical bone formation rate in CKO mice in the unloading model.	^75,76^
Conditional deletion of Cx43 in osteocytes (Dmp1 (10 kb)-Cre; Cx43^*fl/fl*^ CKO)	Cortical bone loss with increased endocortical osteoclast activity during unloading.	^77^
R76W mutation of Cx43	Increased endocortical osteoclast activity and periosteal osteoclasts with decreased apoptotic osteocytes during unloading.	^77^
Conditional deletion of IFT88 in tenocytes (Scx-Cre, IFT88^*fl/fl*^ CKO)	Fibrocartilage cell phenotype in the tendon; thinner and less mineralized cortical bone.	^92^
PIEZO1 KO	Embryonic lethal.	^106^
Conditional deletion of PIEZO1 in osteocytes (OCN-Cre; PIEZO1^*fl/fl*^ CKO)	Disruption of osteogenesis in osteoblasts.	^121^
Conditional deletion of PIEZO1 in mesenchymal stem cells (Prrx1-Cre; PIEZO1^*fl/fl*^ CKO)	Strong hyperactivity of osteoclasts and subtle differences in osteoblastogenesis in PIEZO1-deficient osteoblasts.	^123^
Conditional deletion of PIEZO1 and PIEZO2 in mesenchymal stem cells (Prrx1-Cre; PIEZO1^*fl/fl*^, PIEZO2^*fl/fl*^ DKO)	Severe bone defects.	^125^
Conditional deletion of PIEZO2 in mesenchymal stem cells (Prrx1-Cre; PIEZO2^*fl/fl*^ CKO)	Subtle impact on the bone.	^125^
Conditional deletion of YAP and TAZ in osteoblast progenitors (Osterix-Cre; YAP^*fl/fl*^, TAZ^*fl/fl*^ DKO)	Reduction of the number of osteoblasts and increased osteoclast activity; allele dose-dependent perinatal skeletal deformity.	^188^
YAP KO	Embryonic lethal.	^189^
TAZ KO	Live to maturity with modest skeletal defects and polycystic kidney disease.	^190^
LRP5 G171V transgenic mice	More robust bone formation in response to loading.	^166^
Conditional deletion of BMP2 in osteoblasts (Col1-Cre; BMP2^*fl/fl*^ CKO)	Reduced bone formation rate and increased bone brittleness.	^199^
Conditional deletion of HIF-1α in osteocytes (OC-Cre; HIF-1α^*fl/fl*^ CKO)	Thinner bones with reduced vascularization.	^212^

### The extracellular matrix creates the mechanical niches

The fates and functions of bone cells are determined by the physical and chemical properties of their niches. The niche is a three-dimensional structure consisting of extracellular matrix (ECM) components together with the cells they surround and connect.^28^ The ECM itself is composed of collagens, fibronectin, elastin, laminin, glycosaminoglycan, and glycoproteins. This structure provides a three-dimensional topological environment, an alterable stiffness, and various signaling molecules for the cells.

The mechanical properties of the ECM strongly affect the behavior of cells, including BMSCs, osteoblasts, osteoclasts, and osteocytes. Human mesenchymal stem cells (hMSCs), for instance, can be readily switched from adipogenesis to osteogenesis simply by changing the matrix stiffness.^29^ Osteoblast maturation progressively increases on compact preosteoblast-derived ECM (PDM, a natural self-assembled ECM network), while the same is true for osteoclasts on loose PDM.^30^ This observation also suggests that ECM crosslinking density acts as an underlying force in bone remodeling. Osteocytes are involved in the development and maintenance of the ECM. They are embedded in a continuous matrix and maintain a fluid-filled gap of 50–80 nm between the calcified matrix and the cell membrane; this gap is essential for transporting nutrients and oxygen and generating mechanical signals. Precise regulation of calcification and elongation is also particularly important for osteocytes.^28^ Osteocytes on stiff ECM tend to pull harder than those on soft ECM, thus increasing the tension on force-bearing elements such as F-actin.^31,32^ F-actin serves as both an element of mechanosensing and an effector of mechanotransduction and is the predominant regulator of YAP (Yes-associated protein) and TAZ (transcriptional coactivator with PDZ-binding motif).^33^ These two transcriptional regulators can respond to a broad range of mechanical cues, bridging mechanical signals between the ECM and the cells within the matrix.^34,35^ Accordingly, the ECM constitutes a well-established mechanical niche for bone cells.

### Focal adhesions

**Fig. 3:**
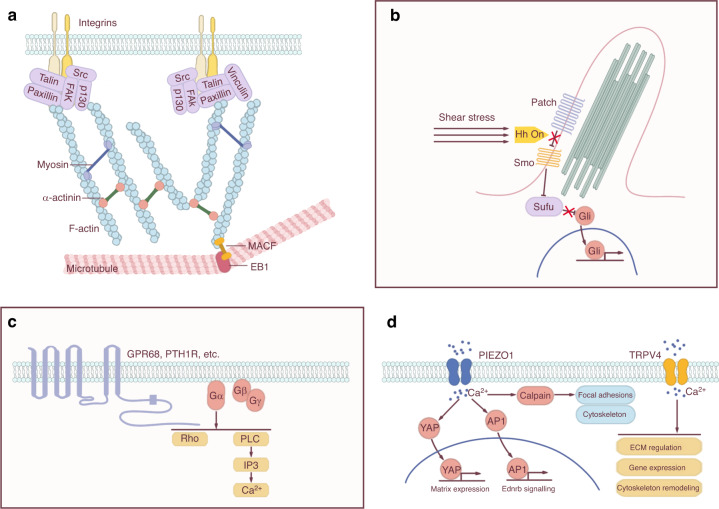
Specific mechanosensitive structures. **a** Focal adhesions. Focal adhesions connect ECM mechanical signals to the cytoskeleton, affecting cytoskeletal arrangement and crosslinking. **b** Cilium. Cilia usually coordinate with the Hedgehog (Hh) signaling pathway to transmit mechanical signals. **c** GPCRs. GPCRs containing a C-terminal helix 8 can sense mechanical stimuli. Activation of the GPCR initiates a series of signal transductions, including the Rho-Rock and PLC-IP3 pathways. **d** Ion channels. Activation of ion channels by mechanical stimuli elicits specific ion flow, especially calcium influx, to modulate downstream signaling pathways.

Focal contacts situated at ECM-integrin junctions form direct mechanical links between cells and the ECM. These structures allow the transfer of signals from the external matrix to the cytoskeleton and facilitate cell adhesion, spreading, and migration. Focal contacts are composed of numerous proteins, including integrins, cadherins, focal adhesion kinase (FAK), and various other ECM and cytoskeletal proteins (Fig. 3a). Their involvement in mechanosensitive signaling pathways highlights the importance of these proteins in skeletal homeostasis.

Integrins are heterodimeric transmembrane receptors consisting of α and β subunits. These molecules functionally link the ECM to the cytoskeleton, thereby transferring mechanical stimuli from the extracellular environment to the internal cellular components. In vertebrates, there are eighteen α and eight β integrin subunits that assemble 24 distinct complexes with diverse nonredundant functions.^36^ Each subunit is a single-span transmembrane glycoprotein composed of a large extracellular segment containing the ligand-binding site, a transmembrane fragment, and a short cytoplasmic domain that mediates interaction with the cytoskeleton or intracellular signaling proteins (except for integrin β4).^37^ Previous studies have suggested that human primary bone cells express multiple integrin subunits, including α1, α2, α3, α4, α5, α6, αv, β1, β3 and β5.^38–40^ The α2, αv, β1, and β3 subunits have been reported to participate in the sensing of mechanical forces.^41,42^

Different integrin heterodimers have specific affinities for ECM ligands such as fibronectin, collagen, and laminin. A series of structural studies have shown that integrins have two allosteric conformations, and their activation is mediated by conformational changes that shift the heterodimer from a low-affinity (inactive) state to a high-affinity (active) state.^43,44^ Integrin aggregation leads to the recruitment and phosphorylation of FAK, which provides a platform for various intermediate proteins (such as MAPK/ERK, JNK, Src kinase, and GTPases) to activate signaling pathways and mediate mechanical signal transduction (Fig. 3a).^45^ For example, blockade of integrin αvβ3 in cultured mouse osteocytes (MLO-Y4 cells) impaired their chemosensitivity to laminar oscillatory fluid flow stimulus, resulting in disrupted COX-2 expression and PGE2 release.^46^ In human osteosarcoma cells (MG-63) cultured on fibronectin, inhibition of β1 or αvβ3 also reduced the shear stress-induced phosphorylation of ERK, JNK, and p38 and upregulated the expression of osteogenic markers.^47^ The activation of ERK by FSS regulated the activity of the RUNX2 transcription factor (associated with osteoblast differentiation) and promoted the expression of β1 via the NF-kB pathway in hMSCs.^48^ Integrins αvβ3 and α2β1 are highly expressed in osteoclasts. They can respond to RGD motif-containing ligands in the bone matrix to regulate osteoclast differentiation and function.^49–51^ However, whether αvβ3 and α2β1 are mechanosensitive in osteoclasts is unknown.

Knockout of integrins leads to embryonic lethality, which limits the in vivo study of bone biology. Integrin β1 conditional knockout mice exhibited a normal phenotype under physiological conditions. However, these mice do not experience bone loss in the hindlimb unloading model compared with wild-type mice; in contrast, they expand both total bone volume and marrow volume and increase whole bone stiffness and strength.^52^ Osteopontin (OPN), which is produced by osteoblasts and osteoclasts, is one of the integrin ligands present in bone. Loss of OPN caused resistance to bone loss in the unloading model. Osteoblasts and osteoclasts were not altered in OPN knockout mice during unloading, suggesting that OPN is a prerequisite for the activation of osteoclastic bone resorption and the reduction in osteoblastic bone formation in this condition.^53^

As mentioned above, FAK is the primary protein that integrates extracellular stimuli with intracellular activities. FAK can sense the mechanical forces generated either internally or externally on cells,^54^ and inactivation of FAK causes defective developmental morphogenesis in mice.^55^ Loss of FAK also disrupted microtubule polarization within cells by impairing focal contact turnover through the FAK-mediated regulation of Rho-family GTPases.^56,57^ Rho-family GTPases are molecular ‘switches’ that control the assembly and disassembly of actin cytoskeletal structures (stress fibers, lamellipodia, and filopodia).^57,58^ RhoA, a member of the Rho family, has been widely implicated in mechanosensitive signaling pathways. ECM stiffness can regulate the activity and expression of RUNX2 through the activation of RhoA/Rho-associated protein kinase (ROCK) and its downstream ERK signaling pathway, thereby promoting osteogenesis.^59^ The RhoA signaling pathway can also activate the Akt and p38/MAPK signaling pathways, establishing a connection between integrins and phosphoinositide 3-kinase (PI3K)/MAPK signaling pathways.^60^

FAK-mediated mechanotransduction in skeletal stem cells activates new bone formation. During mandibular distraction osteogenesis, a surgery that separates the jaw bone to correct an undersized lower jaw, mechanical stress activates new bone formation via FAK in skeletal stem cells.^61^ In osteocytes, sclerostin (*Sost*) can be repressed by mechanical loading through the histone deacetylases HDAC4 and HDAC5.^62^ During this process, FSS triggers FAK inactivation via dephosphorylation, thus driving the nuclear translocation of class IIa HDAC by inhibition of HDAC5 tyrosine 642 phosphorylation.^63^ Similarly, pharmacologic FAK catalytic inhibition reduces *Sost* mRNA expression in vitro and in vivo.^63^ FAK also responds to nonmechanical signals, thus overriding mechanical stimuli. The interaction between bone morphogenetic protein-2 (BMP-2) and BMP-2 receptors activates αvβ3 integrin and mediates cell spreading and cell migration independent of matrix stiffness.^64^ In turn, αvβ3 is required to inhibit glycogen synthase kinase-3β (GSK-3β) activity through the Src–FAK–ILK pathway. BMP receptors and β3 integrin signaling converge to control both focal adhesion dynamics and Smad signaling, coupling cell migration and fate commitment regardless of matrix stiffness.^64^ Relatedly, the bioactive core vitronectin-derived peptide VnP-16 promotes bone formation by accelerating osteoblast differentiation and activity through direct interaction with β1 integrin followed by FAK activation. VnP-16 also inhibits bone resorption by restraining JNK-c-Fos-NFATc1-induced osteoclast differentiation and αvβ3 integrin-c-Src-PYK2-mediated resorptive function.^65^ These investigations address the importance of FAK as a core hub in skeletal mechanobiology.

### Connexons and Cx43

As intercellular channels, gap junctions facilitate signal transduction between cells, allowing small molecules (less than 1 kDa) and electrical current to passively diffuse between adjacent cells in response to extracellular stimuli. Conventional gap junctions consist of two docking hexagonal connexons, both composed of six connexin molecules.^66^ More than 20 connexins with different expression patterns and properties have been discovered in humans and rodents. Diverse connexins are expressed in bone cells, with connexin 43 (Cx43) being one of the most abundant subtypes expressed in osteoblasts, osteocytes, and osteoclasts.^67^

Mechanical stretching can increase the abundance of phosphorylated Cx43 without changing its mRNA expression level in osteoblast-like cells (ROS 17/2.8 cell line).^68^ Another study found that FSS can drive intracellular Cx43 redistribution from a location around the nucleus into the cytoplasm and dendrites. The Cx43 protein level also significantly increased after exposure to FSS from a laminar flow of 16 dynes per cm^2^.^69^ These results indicate that Cx43 has an essential role in sensing mechanical stimuli. In MLO-Y4 cells, conformational activation of integrin α5β1, partially through PI3K activation, allowed it to interact with Cx43. This integrin α5β1 activation is necessary for the mechanical stimulation-induced opening of the Cx43 hemichannel,^19^ which indicates that Cx43 alone cannot sense mechanical stress. These stress-sensing structures are closely coordinated to reinforce cell mechanosensitivity.

Numerous studies have highlighted the importance of Cx43 for normal bone formation, and different Cx43 loss-of-function models have led to different conclusions. *Cx43*^−/−^ mice exhibited osteoblast dysfunction, delayed mineralization, and craniofacial abnormalities.^70^ Conditional deletion of Cx43 by Col1 (2.3 kb)-Cre or Dmp1 (8 kb)-Cre resulted in bone loss and osteoblast function impairment.^71,72^ Regarding mechanical responsiveness, Grimston et al. found that osteoblast-specific Cx43 deficiency driven by Col1 (2.3 kb)-Cre reduces the anabolic response to mechanical loading in vivo.^73^ In contrast, Zhang et al. showed that the Cre-dependent loss of Cx43 in osteocytes/osteoblasts by Ocn-Cre increases osteolytic function via regulation of the RANKL/OPG ratio (a critical determinant of bone mass) and enhancement of the anabolic response to mechanical loading.^74^ Lloyd et al. performed unloading experiments with hindlimb suspension, showing that Cx43 deficiency in osteocytes and osteoblasts protects against trabecular bone loss during mechanical unloading; the effects on cortical bone are more complex, preserving bone formation while potentially allowing relative structural and material changes that ultimately lead to an exaggerated decrease in mechanical properties.^75^ These protective effects of Cx43 deficiency may be due to Sost-mediated suppression of cortical bone formation and decreased cortical osteoclast activity during unloading.^76^ Another research group found that the absence of Cx43 in osteocytes enhances responsiveness to mechanical force in mice.^72^ Their data show that loading induces a greater increase in the periosteal bone formation rate in Cx43 (Dmp1 (8 kb)-Cre) mice than in control mice, resulting in a higher mineralizing surface and enhanced mineral apposition rate.^72^ These researchers further demonstrated that loss of Cx43 in osteocytes promotes the stretch-induced expression of β-catenin and its target genes, thus providing a potential explanation for the increased anabolic response to mechanical signals in mice.^72^ Interestingly, a previous study reported that mechanical stretching increases the abundance of Cx43-specific immunofluorescence staining and protein levels in ROS 17/2.8 cells, increasing gap junctional communication among osteoblastic cells.^68^ Early research also suggested that fluid flow has stimulatory effects on MLO-Y4 cells, encompassing cellular morphology, opening of gap junctions, protein expression, and redistribution of Cx43.^69^ These complex results derived from different systems may be attributable to the dual functions of Cx43 in gap junctions and hemichannels.

Recently, researchers attempted to differentiate the functions of Cx43 in gap junctions and hemichannels in response to mechanical forces using transgenic mice with osteocyte-specific Cx43 deficiency.^77^ The R76W mutation of Cx43 leads to inhibition of gap junctions and enhancement of hemichannels, while the Δ130-136 mutation results in inhibition of both gap junctions and hemichannels. Both mutations cause increased endocortical osteoclast activity during unloading. Increased periosteal osteoclasts and decreased apoptotic osteocytes were observed only in R76W mice.^77^ This study also showed that hindlimb unloading increases empty cortical lacunae of osteocytes in wild-type and Δ130-136 mice compared to R76W mice. Together, these results indicate that the gap junctions of Cx43 are responsible for communication between osteoblasts and osteoclasts, while Cx43 hemichannels protect osteocytes from apoptosis in response to unloading.^77^ In general, it is believed that Cx43 may participate in mechanosensitive signaling pathways and transmit mechanical signals between cells to maintain bone homeostasis during loading or unloading conditions.

### Cilium

The cilium is a microtubule-based antenna-like sensory organelle critical for transducing extracellular mechanical and chemical signals.^78^ It grows from the mother centriole and projects from the cell surface in many vertebrate tissues, including the bone, nervous system, carcinoma, kidney, cartilage, and cardiovascular tissues.^79^ Microtubules form the core of the cilium, also known as the axoneme. Cilia protrude into the extracellular space, where they use unique mechanisms to sense their mechanical environments.^80^

The primary cilium is a potentially important mechanosensitive structure in bone cells^81^ that can sense FSS and mediate mechanotransduction in osteocytes.^82–84^ Cilium formation is positively correlated with the mechanosensitivity of osteocytes; as cilium length increases, osteocyte release of NO and ATP also increases.^85^ Periosteal osteochondroprogenitors directly sense FSS and differentiate into bone-forming osteoblasts via their primary cilia, and this response is almost entirely lost when the primary cilia are absent.^86^ The abrogation of primary cilia in cultured mouse osteoblastic cells (MC3T3-E1 cell line) or MLO-Y4 cells also decreased the normal osteogenic response to FSS.^87,88^ In articular cartilage, the cilia of chondrocytes responded to compression-induced osmotic changes.^89,90^ Indian hedgehog (Ihh) signaling, important in chondrocyte proliferation and differentiation, increased when growth plate chondrocytes were exposed to hydrostatic compression, but this enhanced signaling was abrogated when primary cilia were disrupted (Fig. 3b).^91^ In the tendon enthesis, unloading and overloading led to cilium assembly and disassembly, respectively. Tendon-specific (Scx-Cre) conditional deletion of the ciliary gene *IFT88* caused fibrocartilaginous tendon and weaker mineralized cortical bone through the regulation of hedgehog (Hh) signaling.^92^ The abolition of primary cilia from bone cells also attenuated bone formation in microgravity. Reconstruction of the primary cilia may be a potential strategy for preventing bone loss caused by microgravity.^79,93,94^

### GPCRs

Beyond canonical focal adhesions and cilia, G protein-coupled receptors (GPCRs) have also been proposed as mechanosensitive structures under various physiological and pathological conditions. Only specific GPCRs can sense mechanical forces. Angiotensin II receptor type 1 (AGTR1), for example, can sense mechanical forces to mediate myogenic vasoconstriction,^95^ while bradykinin receptor B2 (BDKRB2) can sense FSS in endothelial cells.^96^ Other mechanosensitive GPCRs include sphingosine 1-phosphate receptors (S1PR), dopamine D5 receptors (D5R), GPR68,^97^ cysteinyl leukotriene 1 receptors (CysLT1R), formyl peptide 1 receptors (FPR1), endothelin ETA receptors, muscarinic M5 receptors, and vasopressin V1A receptors.^98^ Erdogmus et al. found that GPCRs without a C-terminal helix 8 (H8) are not mechanosensitive. Nonsensitive GPCRs can acquire mechanosensitivity by linking an H8 domain, and removing the domain also removes the sensitivity. Moreover, disrupting H8 structural integrity via amino acid mutations impairs the mechanosensitivity it imparts.^98^

Activation of a GPCR via a canonical pathway, such as the binding of its agonist, causes a conformational change that activates its guanine nucleotide exchange (GEF) activity toward one of the possible interacting heterotrimeric Gαβγ proteins (Fig. 3c). This activation results in the replacement of GDP by GTP on the α subunit, causing its activation and dissociation from the βγ subunits. The activated α subunit is classically considered to dissociate from the GPCR, and the αβγ subunits activate different downstream effectors depending on the characteristics of the subunits. For example, αs and αi stimulate and inhibit adenylyl cyclase, respectively; αq can activate phospholipase C, whereas βγ can activate GIRK channels and PI3K. The intrinsic GTPase activity of the Gα subunit causes the system to return to its inactive state, with GDP bound to the Gα subunit of the reconstituted Gαβγ heterotrimer.^99^

Activation of mechanosensitive GPCRs by mechanical stresses can induce downstream signaling events, most notably a phospholipase C (PLC)-IP3- and diacylglycerol (DG)-dependent increase in intracellular calcium concentrations.^100,101^ Mechanosensation occurs alongside chemosensation (the ability to perceive chemicals in the environment) in the intricate physiological environment; thus, cells need to integrate physical and chemical signals appropriately to ensure proper tissue and organ development and function. For example, GPR68 can respond to extracellular acidification during membrane stretching, with its level of activity reflecting both the extent of membrane stretching and the degree of acidification.^102^ There is also evidence that mechanical forces act synergistically with parathyroid hormone PTH (1-34) to regulate bone growth.^103^ GPCRs are critical for the survival and proliferation of skeletal cells in response to surrounding stimuli, and these studies highlight their importance in mechanosensitivity alongside their traditional roles in chemosensation and signal transduction. Further study will be required to expand our understanding of their roles under physiological and pathological conditions in the skeletal system.

### Ion channels

In 2010, Coste et al. identified prominent mechanosensitive cation PIEZO channels (PIEZO1/2) using RNA interference profiles in a mouse neuroblastoma cell line.^104^ Although the PIEZO1 protein shares only 42% sequence homology with its homolog PIEZO2, their structures are similar: a three-bladed, propeller-like trimer with an extracellular cap-like structure embedded in the center and an intracellular beam connecting to the central pore.^105^ In vertebrates, loss of PIEZO1 is lethal, causing malformation of blood vessels during embryonic development.^106^ The conditional loss of PIEZO1 in smooth muscle resulted in hypertension-dependent arterial remodeling dysfunction in mice.^107^ PIEZO1 can regulate the vascular system and lymphatic valve formation^106,108^ in response to fluid shear stress. PIEZOs can also sense blood pressure,^109,110^ dictate neural stem cell differentiation and axon growth in the developing brain by sensing substrate stiffness,^111,112^ and mediate pressure-induced pancreatitis.^113^ In the brain, PIEZO1 distribution is affected by nanotopography, with consequences for hippocampal neuron–astrocyte interactions.^114^ PIEZO2 has essential roles in gentle touch^115^ and proprioception.^116^ It also senses airway stretches and mediates lung inflation-induced apnea.^117^

Bone is very sensitive to mechanical forces, and PIEZO proteins have recently received increasing attention for their potential roles in skeletal mechanosensation. During mandibular arch morphogenesis, PIEZO1 and YAP/TAZ act downstream of *Wnt5a*-mediated cortical polarity and oscillation.^118^ The nuclear abundance of YAP was diminished in the absence of PIEZO1. Interestingly, loss of YAP/TAZ also decreased PIEZO1 expression.^118^ In chondrocytes, PIEZO1 and PIEZO2 can act redundantly to mediate injury-induced osteoarthritis.^119^ In vivo studies have demonstrated the indispensability of PIEZO1 in the osteoblastic lineage, albeit with various phenotypes. Several single-nucleotide polymorphisms (SNPs) of PIEZO1 were associated with osteoporosis and fractures in an atlas of genetic influences on osteoporosis.^120^ Patients with osteoporosis showed reduced levels of PIEZO1 protein, and conditional knockout of PIEZO1 in osteoblast lineage cells disrupted osteogenesis by repressing RUNX2, COL1, and OCN expression.^121^ Two other research groups found increased osteoclast activity in two Cre-induced osteoblastic lineage PIEZO1 knockout systems (Prx1-Cre and Dmp1-Cre).^122,123^ In our own study, we observed very subtle differences in osteogenesis and strong hyperactivity of osteoclasts in osteoblastic PIEZO1-deficient mice.^123^ Our data support the hypothesis that insensitivity to mechanical loading causes dysregulated crosstalk between osteoblasts and osteoclasts, leading to bone loss. These observations also concur with data obtained from space flights, which have little effect on bone formation metabolites but give rise to a strong increase in bone resorption metabolites.^124^ Zhou et al. reported that loss of PIEZO1/2 results in severe bone defects, while the loss of PIEZO2 alone has only a subtle impact on bone; this finding suggests that PIEZO1, but not PIEZO2, is critical for perceiving mechanical forces in bone.^125^ In osteoblastic cells, PIEZO1/2 activates Ca^2+^ influx to stimulate calcineurin, promoting concerted activation of the NFATc1, YAP1, and β-catenin transcription factors in response to mechanical forces.^125^

The transient receptor potential (TRP) multigene superfamily encodes another set of ion channels important in bone mechanosensation. TRP channels are a series of nonselective cation channels composed of integral membrane proteins that can permeate calcium and magnesium ions (Fig. 3d). This superfamily can be divided into 7 subgroups that respond to different stimuli, including mechanical forces.^126^ Among them, TRPV4 serves as a regulator of bone metabolism, a determinant of bone strength, and a potential risk predictor for fractures through the regulation of bone matrix mineralization and intracortical porosity.^127^ TRPV4 can sense mechanical forces in chondrocytes,^128^ bone cells,^129^ epithelial cells,^130^ and endothelial cells.^131^ A previous study reported that FSS in the lacuna activates TRPV4, but not PIEZO1, to increase calcium concentration in the cell plasma.^129^ TRPV4 also mediates oscillatory fluid shear-induced calcium signaling and osteogenic gene (*Cox2* and *Opn*) expression in BMSCs^132^ and plays a critical role in controlling aligned collagen assembly by these cells.^133^ Furthermore, TRPV4 activation in BMSCs accelerates collagen deposition and mineralization.^132^ The expression of late osteogenic genes can also be induced by laminar shear stress through TRPV4.^134^

Recognition of different types and intensities of mechanical stress stimuli is very important for cell mechanotransduction. High-intensity mechanical input is mediated by PIEZO1 and PIEZO2 channels, while low-intensity mechanical input is mediated by TRPV4.^135^ Moreover, TRPV4 tends to be distributed in areas that often have adhesions^136^ or primary ciliary structures.^132^ In BMSCs with defective primary cilia, TRPV4 is insensitive to mechanical activation.^132^ Mutations in TRPV4 are associated with human bone diseases,^137^ and TRPV4 knockout mice showed insensitivity to hind limb unloading.^138^ Loss of TRPV4 in mice also repressed the increased bone resorption during unloading. TRPV4 is additionally expressed in osteoclasts, where it can activate NFATc1 signaling to regulate terminal differentiation and cell activity through Ca^2+^ influx.^139^ Autophagy, which can support osteoclast function, is also regulated by TRPV4.^140^ These data indicate that impairment of TRPV4-mediated osteoclast activity may cause resistance to unloading-induced bone loss.

## Mechanotransduction pathways

Mechanical forces are principal signals that direct cell responses to promote adaptation to the environment. Through mechanosensitive signaling pathways, diverse mechanical stimuli can be translated into biochemical signals that control cell growth, differentiation, apoptosis, and migration. Thus, it is important to understand how various forces and mechanoreceptors are ultimately linked to the activity of nuclear transcription factors. Various references for these mechanosensitive signaling pathways in osteoblastic and osteoclastic lineages are summarized in Table 3.

**Table 3:** References for mechanosensitive signaling pathways in osteoblastic and osteoclastic lineages

Signaling pathways	Osteoblastic lineage	Osteoclastic lineage
Cytoskeleton-integrin	^46–48^	
Cytoskeleton-FAK	^61,63,64^	
Ion channel-Calcium	^125,129,132^	^26^
Cilia-Ihh	^86,87,92^	
Wnt signaling pathway	^161–163^^,^^165,166^	
YAP/TAZ	^118,186,187^	
BMP2 signaling pathway	^198,199^	
Noncoding RNAs	^204–209^	

### Cytoskeleton

The cytoskeleton is a network of fibers formed by the nuclear skeleton, cytoplasmic skeleton, cell membrane skeleton, crosslinking factors, and extracellular matrix. It determines basic cell morphology and connects all mechanosensitive components. Among the cytoskeletal components, actin filament or fibrous actin (F-actin) is well known for perceiving and transmitting mechanical forces in osteocytes.^31^ Previous studies have reviewed the assembly of actin filaments in detail.^141^ A key characteristic of the crosslinked actin network is its ability to stiffen when strained by external or internal forces.^142,143^ Myosin II can guide the assembly patterns of actin filaments and exhibit mechanical stress-dependent kinetics. Myosin II behaves like a crosslinker, stiffening or softening actin networks depending on environmental conditions by modulating filament sliding and rearrangement^144^ or filament disassembly.^145^ This ability to stiffen or soften in response to different intensities of tensile or compressive forces provides cells with intrinsic mechanisms to balance load across heterogeneous structures or to maintain global shape in the face of fluctuating forces. The activity and assembly of myosin II are regulated by the phosphorylation of its light and heavy chains through numerous kinases in diverse regulatory pathways, including myosin light chain kinase (MLCK; activated by Ca^2+^/calmodulin), Rho kinase, citron kinase (activated by RhoA), and myotonic dystrophy kinase-related Cdc-42-binding kinase (MRCK; activated by Cdc-42).^146^ These kinases are all reported to be involved in mechanosensitive signaling pathways.

Microtubule-actin crosslinking factor 1 (MACF1) is a widely expressed cytoskeletal linker that can bind to F-actin and microtubules (Fig. 3a). Coordination between microtubules and F-actin is essential for cell migration and adhesion, which are closely associated with mechanical force transduction. Loss of MACF1 leads to aberrant microtubule organization, tight junction instability, and impaired wound closure in vitro.^147,148^ As MACF1 expression decreases under mechanical unloading conditions in vitro and in vivo, MACF1 is considered a mechanosensitive structure.^149^ This molecule can enhance preosteoblast polarization and migration by raising focal adhesion turnover and end-binding protein 1 (EB1) movement along the microtubule bundle. During this process, MACF1 increases phosphorylated Src (p-Src) levels and promotes colocalization of EB1 with p-Src, resulting in EB1 phosphorylation at Y247; phosphorylated EB1 then moves along the microtubule bundle instead of being anchored at focal adhesion.^150^ Previous studies have also shown that MACF1 significantly promotes mineralization of MC3T3-E1 cells by sustaining the β-catenin/TCF1-RUNX2 axis.^151^

The cytoskeleton determines osteocyte morphology and mechanosensitivity. For example, round osteocytes, which reside within ellipsoid lacunae, seem to have a higher response to mechanical stimuli than more spread, adherent osteocytes.^152^ This finding indicates that osteocytes can sense small strains in their ellipsoid morphology in vivo, thus promoting bone health. Microgravity can cause cytoskeletal depolymerization and changes in the array and direction of microfilaments and microtubules.^153^ In osteoblasts, microgravity induces actin microfilament disruption, thus reducing BMP2-induced activation and translocation of Smad1/5/8 and RUNX2 expression in MC3T3-E1 cells.^154^

**Fig. 4:**
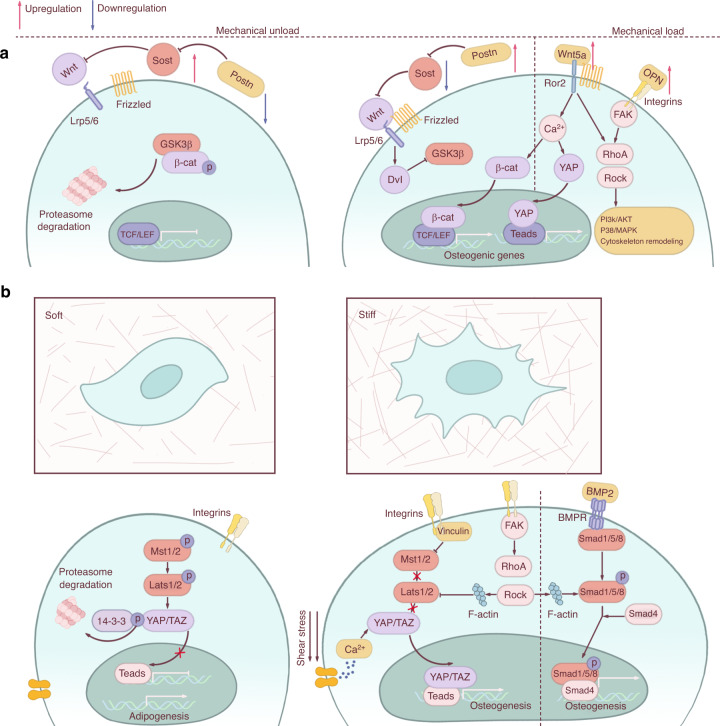
Mechanotransduction signaling pathways. **a** Wnt signaling pathway under mechanical loading and unloading conditions. Unloading can increase the expression of *Sost* and repress the expression of *Postn*, thus inhibiting osteogenic gene expression, while loading has the opposite effect. Wnt and RhoA/Rock/cytoskeleton have synergetic effects in the process of mechanotransduction. **b** MSCs favor a commitment to osteoblasts in the rigid matrix and adipocytes in the soft matrix. Several signaling pathways are involved in this lineage commitment, including cytoskeleton rearrangement, YAP/TAZ nuclear translocation, and BMP2/Smad activation.

The cytoskeleton can transfer mechanical forces, and in turn, these forces can adjust the cytoskeletal structural configuration.^155–157^ Low-intensity vibration (LIV) or mechanical strain can cause a rapid and transient β-catenin association with the nucleoskeleton through the FAK-mediated inhibition of GSK-3β (Fig. 4a). β-catenin molecules that do not bridge cadherins to the actin cytoskeleton or translocate into the nucleus are ubiquitinated and degraded by the 26S proteasome.^158^ Codepletion of the linker of cytoskeleton and nucleoskeleton (LINC) elements Sun-1 and Sun-2 disrupts β-catenin trafficking into the soluble nuclear fraction in response to LIV, thus impairing cytoskeletal integrity and fate determination of BMSCs.^156^

### Wnt/β-catenin signaling pathway

Wnt signaling pathways play diverse roles in skeletal homeostasis.^159^ Canonical Wnt signaling starts when Wnt ligands bind to Frizzled and low-density lipoprotein receptor-related protein 5/6 (Lrp5/6) receptors located on the cell membrane. The activation of Wnt signaling facilitates the accumulation of β-catenin through inhibition of GSK-3β-induced β-catenin phosphorylation, after which dephosphorylated β-catenin translocates into the nucleus to further induce the transcription of LEF/TCF-responsive genes (Fig. 4a).^160^ As a primary intracellular signal transducer for Wnt signaling pathways, β-catenin serves as one of the core hubs for mechanotransduction.^156^

Wnt activity is increased by mechanical loading and decreased by unloading. Mechanical loading activates the β-catenin signaling pathway through a mechanism involving nitric oxide, FAK, and the Akt signaling pathway.^161^ Mechanical strain induces MSCs away from adipogenic differentiation and toward osteogenic differentiation through preservation of β-catenin in the nucleus.^162^ Pulsed electromagnetic fields (PEMFs), an effective method for healing fractures and improving osteoporosis, can induce osteogenesis by increasing intracellular Ca^2+^, which in turn leads to nucleoplasm translocation of β-catenin in C3H10T1/2 mesenchymal cells.^163^

Osteocytes also appear to use the Wnt/β-catenin pathway to transmit mechanical loading signals into cells. Strength and power training, leveraged clinically to enhance bone quality, upregulated the expression of Wnt-related genes in humans.^164^ Mice lacking β-catenin in osteocytes showed severe osteopenia and skeletal fragility and died prematurely. β-catenin heterozygous mice also had impaired responses to mechanical loading.^165^ Osteoblasts derived from LRP5 G171V (Wnt signaling activation) transgenic mice exhibited increased expression of Wnt/β-catenin target genes under physiological conditions. Moreover, LRP5 G171V transgenic mice showed more robust bone formation in response to loading, with an elevated expression of genes downstream of the Wnt signaling pathway, than wild-type mice.^166^ Similarly, the anabolic response of bone to mechanical loading was enhanced in the ulnae of mice lacking the Wnt inhibitor FRZB.^167^ In mice deficient in Sost, an extracellular Wnt signaling antagonist, bone formation was accelerated, especially around the periosteum in the setting of cyclic axial loading (Fig. 4a).^168^ Periostin (Postn), an ECM protein with important roles in osteogenesis, is highly expressed in the bone periosteum. Mechanical loading specifically reduces Sost expression and increases Postn expression, while the opposite is true for unloading.^169,170^ Loss of Postn in mice caused bone loss, which was reversed by an anti-Sost antibody. In vitro studies further showed that Postn can suppress Sost expression through the integrin αVβ3 receptor (Fig. 4a).^171^ Together, these data support the hypothesis that Wnt/β-catenin signaling is a physiological response to loading and that activation of the Wnt/β-catenin pathway enhances the sensitivity of osteoblasts/osteocytes to mechanical loading.

Noncanonical Wnt signaling can also be elicited by mechanical forces. Oscillatory fluid flow can increase the expression of Wnt5a and its noncanonical tyrosine kinase receptor Ror2, both of which are necessary for mechanically stimulated RhoA activation, ultimately resulting in osteogenesis (Fig. 4a).^172^ Overexpression of Ror2 has also been reported to enhance osteogenesis.^173^ These studies provide evidence that noncanonical Wnt signaling pathways also participate in mechanotransduction.

Previous studies have also addressed the importance of interconnections between different signaling pathways. Of note, high-magnitude mechanical stress can inhibit canonical Wnt signaling-induced osteoblastic differentiation. In this case, large-magnitude loading exhibits an inhibitory effect on the PI3K/Akt pathway, resulting in the accumulation of GSK-3β and phosphorylation of β-catenin.^174^ Inhibition of FAK or PI3K can abolish the fluid flow-induced stabilization of β-catenin and consequently inactivate the Wnt/β-catenin pathway in osteocytes.^161^

### YAP/TAZ

The Hippo pathway integrates a broad range of signals to control key cellular processes through the activity of YAP and TAZ.^175^ YAP and TAZ are transcriptional coregulators that do not contain DNA-binding domains; thus, they usually interact with DNA-binding proteins to regulate transcriptional activity. Once activated, the Hippo pathway limits tissue growth and cell proliferation by phosphorylating and inhibiting YAP/TAZ. In mammals, MST1/2 form heterodimers with SAV1 through their C-terminal SARAH domains, after which MST1/2 can phosphorylate SAV1, MOB1, and LATS1/2 kinase.^176,177^ LATS1/2 directly phosphorylates YAP and TAZ at multiple sites, thereby inhibiting their nuclear localization.^178^ Phosphorylated YAP/TAZ is sequestered in the cytoplasm by interaction with the 14–3–3 protein and then degraded by the ubiquitin-proteasome system (Fig. 4b).^179^ Alternatively, when the Hippo pathway is off, YAP/TAZ are dephosphorylated and translocate into the nucleus, where they bind to cotranscriptional factors to induce transcriptional programs important for cell proliferation, survival, and migration.^175,180–182^

Several upstream inputs have been reported to regulate the nuclear localization of YAP/TAZ in response to mechanical stresses. In luminal breast cancer MCF7 cells, RAP2, a Ras-related GTPase, was activated by a soft matrix, relaying ECM rigidity signals to control mechanosensitive cellular activities through YAP/TAZ. In this model, low stiffness acts through phospholipase Cγ1 (PLCγ1) to increase the levels of phosphatidylinositol 4,5-bisphosphate and phosphatidic acid, which activate RAP2 through PDZGEF1 and PDZGEF2. Finally, RAP2 can activate MAP4K4, MAP4K6, MAP4K7, and ARHGAP29 through phosphorylation, resulting in activation of LATS1 and LATS2 and inhibition of YAP and TAZ.^183^ The SWI/SNF chromatin-remodeling complex can interact directly with purified F-actin^184^ and inhibit YAP/TAZ through ARID1A in response to mechanical stiffness.^185^

YAP/TAZ are also functionally required for the differentiation of MSCs in response to ECM stiffness; conversely, the expression of activated YAP overrules physical constraints in dictating cell behaviors.^186^ Rigid stiffness increases the abundance of cytoskeleton-associated vinculin. This vinculin then promotes the nuclear transportation of YAP/TAZ independent of LATS1, contributing to the regulation of ECM stiffness-dependent adipocyte differentiation of MSCs.^187^ Loss of both YAP and TAZ in osteoblastic lineage cells destroys the integrity of the skeletal system, reducing the bone formation and elevating bone resorption.^188^ The convergence of these different pathways on the regulation of YAP/TAZ nuclear localization, whether dependent or not on the Hippo pathway, reflects the diversity of upstream inputs and efficiency of biological responses to the environment.

Although YAP and TAZ are thought to be largely redundant and similarly regulated by Hippo signaling, both of them have specific structural, developmental, and physiological characteristics. Notably, global YAP deletion in mice was embryonic lethal due to impaired yolk sac vasculogenesis,^189^ whereas global TAZ knockout mice survived to maturity with only modest skeletal defects and polycystic kidney disease.^190^ This evidence shows that YAP and TAZ have exclusive, gene-specific functions. The exact function of YAP in osteogenesis remains controversial in vitro. YAP has been reported to suppress osteogenesis through the repression of RUNX2 transcriptional activity in response to Src family kinases.^191^ In contrast, a separate study found that overexpression of a constitutively active YAP mutant in MSCs promotes osteogenesis more than adipogenesis, even under soft matrix conditions.^186^ Additionally, YAP overexpression has been reported to inhibit both the osteogenic and adipogenic abilities of MSCs through inhibition of Wnt/β-catenin signaling by interfering with β-catenin and inducing Dkk1 expression.^192^

TAZ was reported to contribute to osteogenic differentiation by modulating the canonical Wnt pathway.^193,194^ TAZ can also promote osteogenesis and inhibit adipogenesis by acting as a RUNX2 coactivator and PPARγ inhibitor.^195^ In vivo, osteoblast-specific overexpression of TAZ promoted bone formation with higher expression of RUNX2.^196^ Recently, a mouse model of combinatorial YAP/TAZ ablation in skeletal lineage cells (Osterix-Cre) showed allele dose-dependent perinatal skeletal deformity, demonstrating that YAP and TAZ have concordant functions in osteoblast lineage cells.^188^ YAP/TAZ depletion and acute YAP/TAZ–TEAD inhibition reduced osteogenic and collagen-related gene expression.^188^ Our previous data demonstrated that YAP serves as the downstream element of PIEZO1 that regulates matrix protein expression.^123^ The components of the extracellular matrix determine the rigidity of the ECM, which in turn affects the nuclear localization of YAP/TAZ. This feedback implies that YAP/TAZ can modulate the ECM in response to mechanical forces, contributing to the delicate process of adaptation between the ECM and cells.

### BMP2 signaling pathway

Bone morphogenetic protein 2 (BMP2) belongs to the transforming growth factor β (TGFβ) superfamily and participates in bone, cartilage, and joint formation. The BMP2 signaling pathway is currently a major therapeutic target for improving bone mass or quality, ameliorating skeletal overgrowth diseases, and repairing damage to bones and joints. BMP2 binds to and activates BMP transmembrane receptors I (BMPR-IA) and II (BMPR-II), after which activated BMPR-IA transmits signals via recruitment and phosphorylation of R-Smads, including Smad1, Smad5, and Smad8. R-Smads subsequently forms a complex with common Smads (Smad4) and translocate into the nucleus to activate the transcription of target genes such as *Runx2* and *Alp* (Fig. 4b).^197^ Alternatively, BMP2 signaling can be mediated by the TAK1-p38 kinase pathway.^198^ BMP2 deficiency in osteoblasts results in osteopenia and bone strength reduction.^199^

BMP2 is also involved in mechanotransduction. During the commitment of hMSCs toward the osteogenic lineage, cell shape can modulate the ability of BMP2 to activate RhoA, ROCK, and cytoskeletal tension. In turn, RhoA/ROCK activity and associated cytoskeletal tension regulate hMSC commitment to the BMP-induced osteogenic phenotype.^200^ The osteogenesis induced by BMP2 also depends on matrix stiffness. On soft matrix (0.5–3.5 kPa), hMSCs cannot elicit BMP2-induced osteogenesis.^201^ Proper organization of the F-actin cytoskeleton in response to the microenvironment’s mechanical properties is necessary for BMP2-induced Smad1/5/8 phosphorylation and nuclear translocation.^201^ Studies have shown that mechanical loading can enhance the ability of transplanted mesenchymal condensations containing BMP-2 and TGF-β1 to regenerate bone in vivo,^202^ indicating that interacting chemical and physical factors in ECM scaffolds may work synergistically to enhance bone regeneration.

BMP2 can also act in coordination with other mechanosensitive signaling pathways. For example, the myoblast C2C12 cell line can undergo osteogenesis in the presence of both stiff matrix and the BMP2 signaling pathway. Without BMP2, C2C12 cells are unable to differentiate into osteoblasts regardless of matrix stiffness due to the inactivation of Smad signaling. The initiation of BMP2-induced Smad signaling is independent of cytoskeletal tension in these cells.^203^ However, osteogenic gene activation requires cytoskeletal tension-induced nuclear accumulation of YAP/TAZ, which depends on matrix stiffness (Fig. 4b).^203^ Thus, osteogenesis in C2C12 cells may be under the control of synergistic cooperation between multiple transcription factors and the mechanical environment.

### Noncoding RNAs (lncRNA and miRNA)

Emerging evidence has revealed that noncoding RNAs possess the ability to transmit mechanical signals. Among the noncoding RNAs, miRNAs specifically have been reported to mediate mechanical force transduction during osteogenesis.^204^ Under simulated microgravity conditions, for example, miR-139-3p and the lncRNA ODSM coordinately regulated the differentiation and apoptosis of MC3T3-E1 cells,^205^ while the miR-30 family (except miR-30a) inhibited their osteoblastic differentiation.^206^ miR-33-5p can promote osteogenesis in vitro; osteoblast-targeted delivery of this miRNA in mice partially counteracted the reduction of osteogenic genes and mineral apposition rate in the hindlimb unloading model.^207^ Clinically, increased levels of miR-138-5p were detected in bone specimens from bedridden and aged patients with a lower degree of bone formation. Inhibition of this miRNA in the bone rescued the reduced bone mass in the hindlimb unloading model and enhanced the bone anabolic response during exercise. Further studies showed that miR-138-5p can directly target MACF1 to inhibit osteoblast differentiation.^208^ Both miR-100-5p and miR-143-3p significantly enhanced osteogenesis in a 3D soft hydrogel.^209^ These data indicate that modulation of miRNA signaling and substrate stiffness can direct MSC lineage commitment and provide a novel approach for bone regeneration.

### Potential factors and pathways

In addition to the molecules and pathways mentioned above, various other transcription factors contribute to mechanotransduction. In myeloid cells, PIEZO1 can respond to cyclical hydrostatic pressure and drive c-JUN activation and transcriptional upregulation of endothelin-1 (EDN1). This change in turn stabilizes HIF1α, thus facilitating the transcription of proinflammatory genes.^210^ Mechanical forces can also activate Ras/ERK-mediated mitogen-activated protein kinase (MAPK) signaling, promoting HIF-1α expression in osteoblasts.^211^ Osteoblast-specific deletion of HIF-1α in mice resulted in thinner bones with reduced vascularization, whereas deletion of the E3 ligase von Hippel-Lindau protein (pVHL) promoted bone vascularization.^212^ Further studies will undoubtedly continue to reveal transcription factors involved in metabolic and hypoxic modulation in response to mechanical forces.

## Bone remodeling induced by microgravity

Previous studies have shown that bone loss in astronauts is caused by apoptosis of osteocytes and increased bone resorption.^213–216^ Both osteoclasts and osteoblasts can respond to mechanical stimulation in vitro. Nevertheless, the precise contributions of bone formation and resorption to microgravity-induced bone loss remain to be fully elucidated.^214,217,218^ During space flight, increased bone resorption results in the loss of calcium and minerals in the bone, altering the endocrine regulation of calcium metabolism.^124,214^ Based on these observations, osteoclasts might be presumed to modulate bone quality by sensing mechanical forces directly or by receiving signals from osteoblastic lineage cells. An in vitro study demonstrated that the duration and amplitude of mechanical loading determine the directionality of its effect on the osteoclastogenesis of hematopoietic progenitor cells,^27^ suggesting that osteoclasts have the potential to sense mechanical forces. Nevertheless, in most studies, osteoclasts are more likely to be effectors that accept signals from other mechanosensitive cells and execute tasks. Thus, osteoclasts are both mechanosensitive cells and effectors for other mechanosensitive cells. Mesenchymal stem cells or osteocytes exposed to oscillatory fluid flow could significantly inhibit osteoclastogenesis in a coculture system.^219,220^ In vivo, monocytes, the progenitors of bone-resorbing osteoclasts, were recruited by CXCL1 and CCL2 produced by mesenchymal stem cells treated with mechanical forces.^221^ Our recent study found that loss of PIEZO1 in osteoblastic lineage cells leads to a deterioration of the bone structure and quality due to increased bone resorption, and accelerated osteoclast formation is determined by the altered bone matrix secreted by osteoblastic cells.^123^ It is not just the autonomous activity of osteoclasts but also the changing niches or factors originating from other cells that affect the function of osteoclasts. These investigations provide a mechanism for the increased bone resorption observed in microgravity environments.

## Concluding remarks and future perspectives

Since the 19^th^ century, the correlation between mechanical forces and bone mass has been of great interest in the field of skeletal mechanobiology.^1^ Although the mechanosensitive signaling pathways in the skeleton are diverse, they are inseparably linked with each other. Future studies should address several questions. First, the mechanical environment of skeletal cells in vivo is highly complex. Mesenchymal stem cells and their progenies, including osteoblasts and osteocytes, receive diverse stress stimuli with correspondingly varied outcomes. The precise correlation between the types of mechanical stresses and their downstream signaling pathways should be further explored by mimicking the stress environment in vitro with the help of 3D culture and bone biomimetic materials. Second, different exercise styles have distinct influences on the skeletal system. The intensity and type of workout may lead to different results. Proper exercise can increase bone mass, while increasing exercise intensity to excessive levels can diminish bone mass and quality and even cause increased stress fractures.^222,223^ Consequently, studies using physical therapy must ensure that the effects of intensity and style of exercise can be properly inferred. Further investigation into the potentially harmful effects and the downstream responding factors of overload is also warranted. Finally, as older people gradually lose the capacity to exercise, the reduction of stress stimuli over time may contribute to bone loss. Effective treatment strategies should first establish the underlying mechanisms that drive these changes in cell physiology and then design therapies directed toward these mechanisms.

An important factor that limits the field of skeletal mechanobiology is the lack of devices suitable for studying mechanosensitive signaling pathways. In most previous studies, cell lines have been subjected to unidirectional or oscillatory fluid flow.^224^ Cell lines can also be seeded in a culture dish with a retractable bottom to apply tension and squeezing force^225^ or in a gel to apply compression force.^226^ Such conditions may not reflect the in vivo mechanical environment, and most of the present studies are based on observations in 2D systems. Some 3D structures or scaffolds, including multivacancy structures made of collagen, hydroxyapatite substrates, and synthetic materials, which partially simulate the physiological environment, are used in bone cell research. Simulated microgravity is achieved by slow-rotating wall vessels (SRWVs), random positioning machines (RPMs), and 3D clinostats,^227,228^ mainly based on a rotating fluid environment. In animal models, mechanical loading and unloading can be achieved by cyclic pressure on long bones, different exercise devices, and tail suspension.^229,230^ Nevertheless, these models alone are not enough to ultimately determine the changes in cell morphology, genes, and hormones in a weightless environment. The true microgravity environment relies on the support of the International Space Station. Recently, several studies on spaceflight have used systemic analysis to clarify changes in multiple dimensions from metabolism to genetics or epigenetics in various tissues and cells. These studies provide new insights into the environment of outer space, including radiation and microgravity, and the way it affects biological and physiological states.^231–234^ Support from the aerospace agency will continue to help uncover more solid, physiologically accurate findings on skeletal mechanobiology.
